# Incidental cardiac findings on somatostatin receptor PET/CT: What do they indicate and are they of clinical relevance?

**DOI:** 10.1007/s12350-021-02526-9

**Published:** 2021-01-27

**Authors:** Emanuele Bobbio, Anna Dudás, Anders Bergström, Daniela Esposito, Oskar Angerås, Amar Taha, Martijn van Essen, Marie Björkenstam, Kristjan Karason, Entela Bollano, Niklas Bergh, Christian L. Polte

**Affiliations:** 1 Department of Cardiology, Sahlgrenska University Hospital, Gothenburg, Sweden; 2 Department of Clinical Physiology, Sahlgrenska University Hospital, Gothenburg, Sweden; 3 Department of Clinical pathology, Sahlgrenska University Hospital, Gothenburg, Sweden; 4 Department of Endocrinology, Sahlgrenska University Hospital, Gothenburg, Sweden; 5 Department of Transplantation, Sahlgrenska University Hospital, Gothenburg, Sweden; 6 Department of Radiology, Sahlgrenska University Hospital, Gothenburg, Sweden; 7 Institute of Medicine, The Sahlgrenska Academy at the University of Gothenburg, Gothenburg, Sweden; 8 Institute of Biomedicine, The Sahlgrenska Academy at the University of Gothenburg, Gothenburg, Sweden

**Keywords:** Neuroendocrine tumor, pheochromocytoma, myocardial infarction, inflammation, somatostatin receptor, 68Ga-DOTATOC, positron emission tomography

## Abstract

We present the case of a 47-year-old man with a history of recurrent episodes of frontal headache, fever, and chest discomfort as well as longstanding, difficult to treat arterial hypertension. Clinical work-up revealed the unexpected finding of an underlying pheochromocytoma as well as recent “silent” myocardial infarction. Our case highlights the importance of paying attention to incidental cardiac findings on somatostatin receptor positron emission tomography/computed tomography, as routinely performed in patients with clinically suspected neuroendocrine tumors. These incidental cardiac findings cannot only indicate a primary or secondary (metastatic) neuroendocrine tumor, but also areas of myocardial inflammation, as somatostatin receptors cannot only be found on the majority of neuroendocrine tumors, but also among other tissues on the surface of activated macrophages and lymphocytes. The detection of myocardial inflammation is of clinical importance and its underlying etiology should be evaluated to prompt eventual necessary treatment, as it is a potential driving force for cardiac remodeling and poor prognosis.

## Introduction

Incidental imaging findings are a fast growing entity due to the overall rapid rise in demand for advanced medical imaging in combination with the continuously ongoing technical and medical progress, which results in new and potentially better diagnostic abilities. In clinical routine, these incidental findings pose quite a challenge, as they might be of clinical relevance or just lead to a cascade of unnecessary and potentially harmful studies to follow.^1^,^2^

Little is known about how to manage incidental cardiac findings on somatostatin receptor positron emission tomography/computed tomography (PET/CT), as routinely performed in patients with clinically suspected neuroendocrine tumors (NETs).^3^ Here, we describe a case that sheds light on what these imaging findings might indicate and if they are of any clinical relevance.

## Case Report

**Figure 1:**
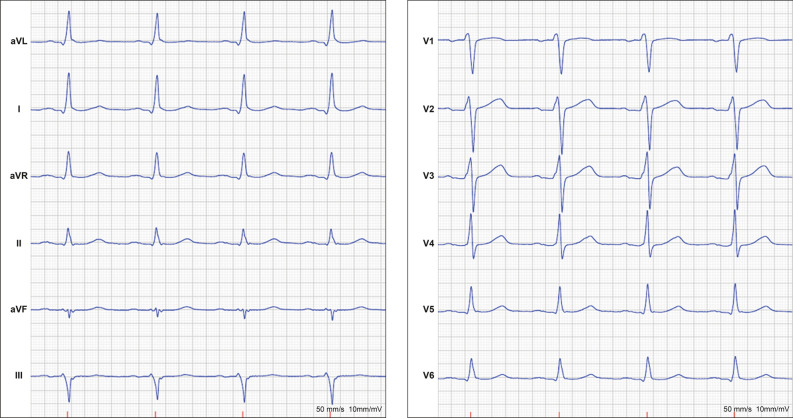
Normal resting electrocardiogram without signs of ischemia or prior myocardial infarction

**Figure 2:**
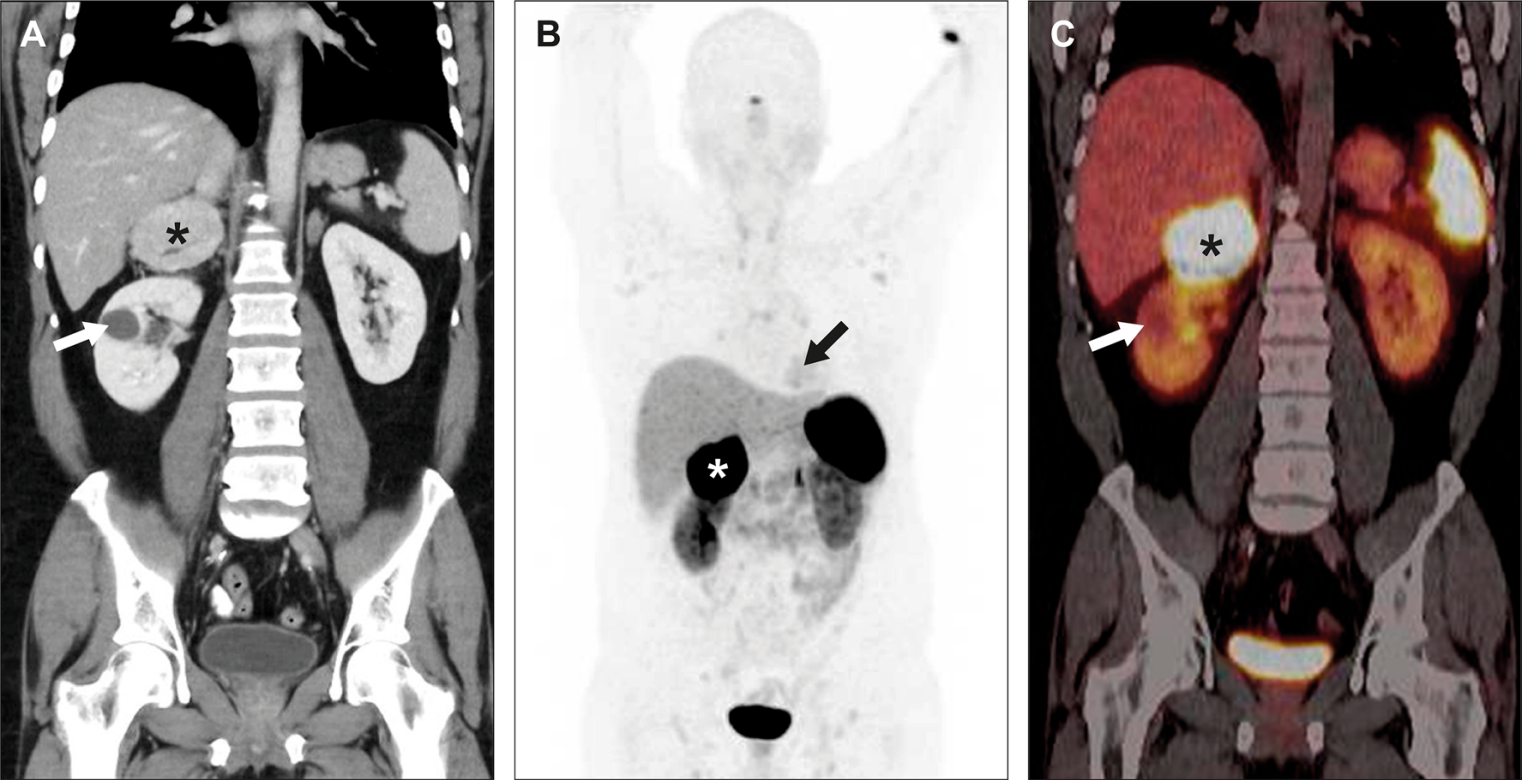
Abdominal computed tomography (**A**) revealed a large (approximately 7 × 6 × 6 cm) right-sided adrenal mass with central necrosis (as indicated by a black star), which showed clearly pathologic uptake on ^68^Ga-DOTATOC PET/CT (as indicated by a white star in the maximum intensity projection image (**B**) and a black star in the PET/CT fusion image (**C**)). Additionally, the maximum intensity projection image (**B**) displayed a low cardiac uptake (as indicated by a black arrow). Furthermore, a renal cyst was found in the right kidney (as indicated by a white arrow (**A** and **C**)). Maximum standardized uptake values: liver 7.5, spleen 35.3, adrenal mass 135.9, and heart 5.0

**Figure 3:**
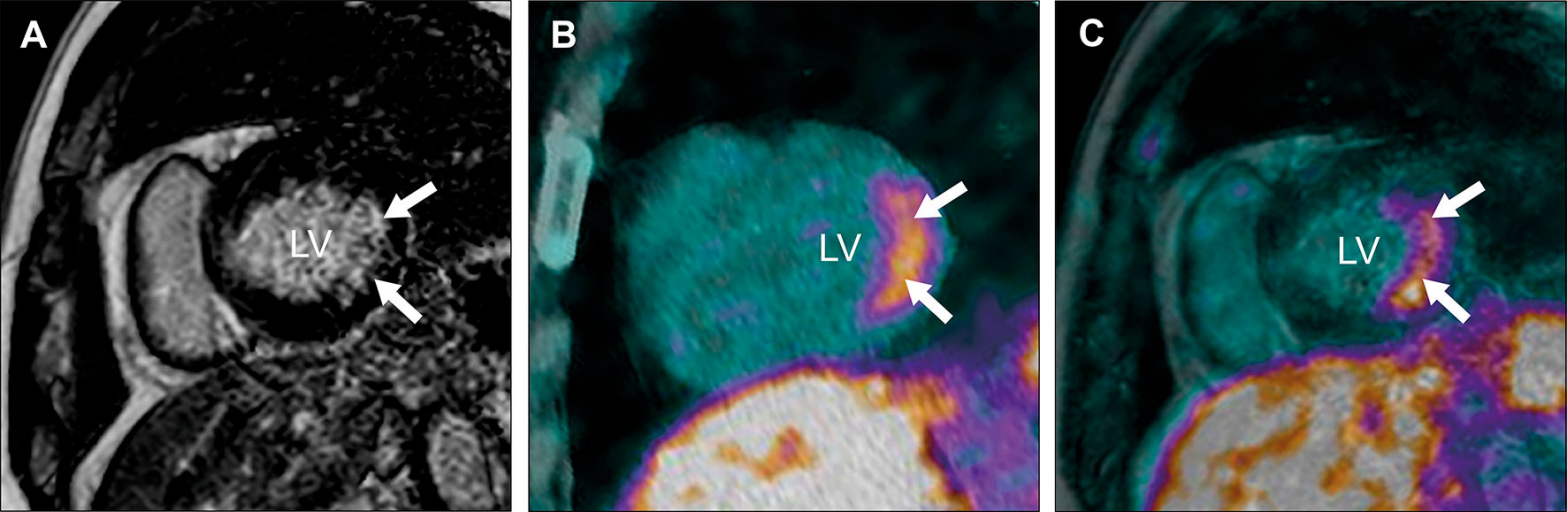
CMR revealed the presence of inferolateral subendocardial delayed enhancement in the basal part of the left ventricle (LV) in the short-axis projection (**A**). In the same region, ^68^Ga-DOTA-TOC PET/CT showed pathologic myocardial uptake (**B**). Image fusion of the ^68^Ga-DOTA-TOC PET and CMR confirmed the colocalization of both findings (**C**). White arrows indicate pathologic findings

**Figure 4:**
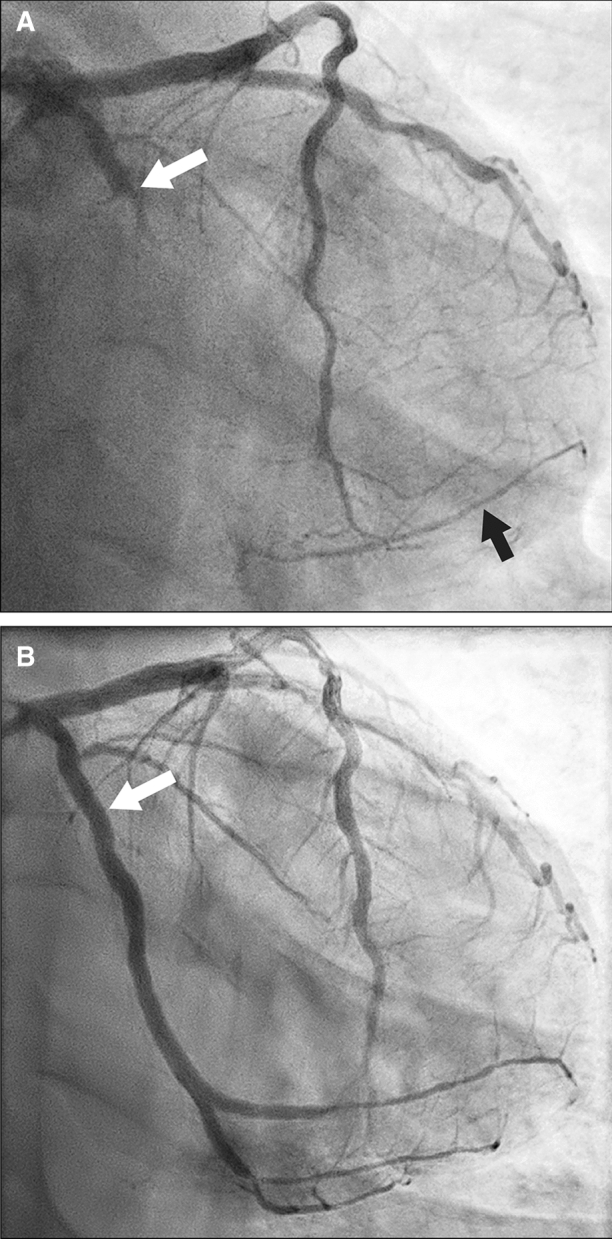
Invasive coronary angiography (**A**) unveiled an occluded left circumflex artery (as indicated by a white arrow) with collateral flow in two obtuse marginal branches (as indicated by a black arrow). Furthermore, a borderline significant stenosis was found in the left anterior descending artery as well as a high degree stenosis in the right coronary artery (not shown). Subsequently, percutaneous transluminal coronary angioplasty with stent implantation could, among others, successfully reopen the left circumflex artery (as indicated by a white arrow, **B**)

**Figure 5:**
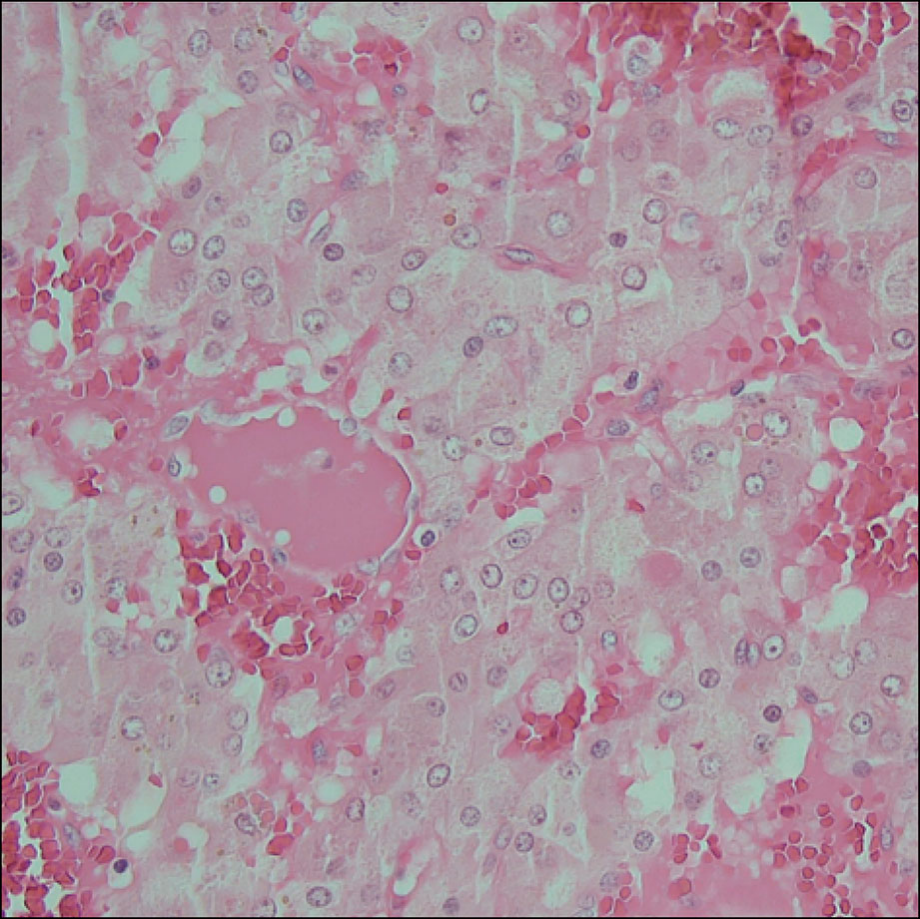
Classic histopathologic appearance of a pheochromocytoma with large polygonal cells arranged in small nests (so-called Zellballen), which are separated by capillaries filled with erythrocytes (hematoxylin and eosin staining)

A 47-year-old man from Syria was admitted to a regional Swedish hospital due to a three-month history of recurrent episodes of frontal headache and fever. Otherwise, he showed no further infection-related symptoms. The past medical history revealed longstanding problems (approximately for the last 20 years) with difficult to treat arterial hypertension, where numerous medications had been tested without success or withdrawn due to side effects. No previous work-up concerning eventual secondary hypertension had been undertaken. Additionally, the patient experienced recurrent episodes of chest discomfort in relation with food intake during the last nine months, which was empirically treated with proton-pump inhibitors without success. Upon admission, the patient had a blood pressure of 160/100 mmHg, an initial heart rate of 99 beats per minute, a saturation of 96%, and an elevated body temperature of 40° Celsius. Otherwise, the initial physical exam was unremarkable. The electrocardiogram showed regular sinus rhythm without ST-T segment alterations or Q-waves (Figure 1) and the initial blood analysis was normal (including the liver and thyroid function) apart from an elevated C-reactive protein (Table 1). Subsequently, blood and urine cultures were taken, which were all negative. A serum and urine electrophoresis showed normal findings as well as a lumbar puncture. Echocardiography showed no signs of infective endocarditis. Additionally, both thoracic and abdominal computed tomography were performed in search of the origin of the patient’s recurrent fever, which revealed a right-sided adrenal mass with central necrosis as well as a renal cyst (Figure 2A). Consecutive endocrinological investigations according to current guidelines substantiated the suspicion of an underlying pheochromocytoma (Table 1).^4^ The initial symptoms upon presentation as well as the increase in C-reactive protein were interpreted as secondary to a suspected viral infection. In the following, the patient was referred to the regional University hospital for further diagnostic work-up and treatment. Subsequently, the patient underwent a Gallium-68 (^68^Ga) DOTATOC PET/CT, which revealed high uptake in the right-sided adrenal mass consistent with a pheochromocytoma (Figures 2B and C). Additionally, low cardiac uptake was incidentally discovered in the basal inferolateral segment of the left ventricle (Figures 2B and 3B). This uptake of low intensity was interpreted as a suspected area with myocardial inflammation due to recent myocardial infarction or myocarditis. A primary or secondary (metastatic) cardiac NET was considered as less likely. In the light of these findings, a new thorough medical history revealed that the patient’s recurrent episodes of chest discomfort had even a relation to physical activity and not only food intake. This made the diagnosis of an underlying coronary artery disease even more likely together with the presence of coronary calcification on the previously performed thoracic computed tomography. Furthermore, the patient had several cardiovascular risk factors such as longstanding arterial hypertension, smoking (approximately 10 cigarettes per day for at least 20 years) and a positive family history for cardiovascular disease. To further determine the underlying etiology of the incidental cardiac finding, a complementary cardiovascular magnetic resonance (CMR) study was performed, which revealed in the same region subendocardial delayed enhancement consistent with an ischemic etiology as well as regional hypokinesia (Figures 3A and C). T2-weighted images showed no signs of edema. Furthermore, CMR revealed mild concentric left ventricular hypertrophy and a reduced left ventricular ejection fraction of 41%. Taken together, the presence of myocardial inflammation on ^68^Ga-DOTATOC PET/CT and subendocardial delayed enhancement as well as regional wall motion abnormality on CMR suggested that the patient had most likely suffered of a recent “silent” myocardial infarction (no recent clinical symptoms of persistent or worsening chest pain). Therefore, the patient underwent an invasive coronary angiography that unveiled the presence of a multi-vessel coronary artery disease including an occluded left circumflex artery, which supplies the inferolateral segments of the left ventricle with blood (Figure 4A). Subsequently, a percutaneous transluminal coronary angioplasty with stent implantation was performed that could, among others, successfully reopen the left circumflex artery (Figure 4B). Afterwards, medical treatment was optimized according to current guidelines and the patient was relieved of his previous recurrent episodes of chest discomfort. Furthermore, the patient underwent an uncomplicated right-sided laparoscopic adrenalectomy prior to the earlier mentioned invasive coronary angiography, which resulted in the resolution of his previous symptoms as well as normalization of the plasma free normetanephrine concentration (Table 1). This strategy was favored as the patient was clinically stable, the perioperative risk considered as acceptable and to avoid the dilemma arising from dual antiplatelet therapy preceding non-cardiac surgery. Routine histopathologic analysis of the removed adrenal mass revealed a classic appearance of a pheochromocytoma (Figure 5).

**Table 1:** Laboratory results

	Initial work-up	Post-surgical follow-up	Reference values
Hematology and blood chemistry			
WBC, 10^9^/L	8,7	–	3.5–8.8
RBC, 10^12^/L	4,7	–	4.2–5.7
Hb, g/L	131	–	134–170
Platelets, 10^9^/L	297	–	145–348
Creatinine, µmol/L	63	–	60–105
Sodium, mmol/L	133	–	137–145
Potassium, mmol/L	3.7	–	3.5–4.4
C-reactive protein, mg/L	79	–	< 5
Endocrinological investigations			
Plasma			
Metanephrine, nmol/L	0,7	0.2	< 0.5
Normetanephrine, nmol/L	57	0.6	< 1.1
Chromogranin A, µg/L	940	–	< 102
Aldosterone/renin ratio, pmol/mIE	0.7	–	4–65
Cortisol after DST, nmol/L	370	–	133–537
Urine			
Urinary adrenalin, nmol/24 hours	23	–	9–101
Urinary noradrenalin, nmol/24 hours	799	–	62–560
Urinary metanephrine/creatinine ratio, mmol/mol creatinine	0.1	–	–
Urinary normetanephrine/creatinine ratio, mmol/mol creatinine	6.7	–	–
5-HIAA, µmol/24 hours	21	–	0–50

*WBC*, white blood cell count; *RBC*, red blood cell count; *Hb*, hemoglobin; *DST*, dexamethasone suppression test; *5-HIAA*, 5-hydroxyindoleacetic acid

## Discussion

Expression of somatostatin receptors, which belong to the superfamily of G-protein coupled receptors and constitute of 5 subtypes, cannot only be found on the majority of NETs but also among other tissues on the surface of activated macrophages and lymphocytes (specifically somatostatin receptor subtype-2 and subtype-3).^5^,^6^ Therefore, it is not surprising that a small number of previous studies were able to show the ability of somatostatin receptor PET/CT to visualize both vascular as well as cardiac inflammation.^6^,^7^ In contrast to Fluor-18 fluorodeoxyglucose PET/CT with its high background signal, low leukocyte specificity, and need for rather complicated preparation techniques to suppress physiological myocardial glucose uptake, somatostatin receptor PET/CT targets inflammatory cells directly and does not have to cope with relevant physiological cardiac uptake. This seems to be a clear diagnostic advantage.

Myocardial inflammation can be due to a large variety of underlying etiologies, ranging from myocarditis to post-ischemic inflammation or different forms of cardiomyopathy, and is a potential driving force for ventricular remodeling and poor prognosis.^8^–^10^ In our case, the observed myocardial inflammation was due to a recent “silent” myocardial infarction, as diagnosed by a combination of ^68^Ga-DOTATOC PET/CT and CMR. Myocardial infarction initiates a characteristic time-dependent inflammatory cascade that constitutes of three overlapping phases, namely an inflammatory, a reparative/proliferative and a healing phase, resulting in the formation of a mature scar within 4-6 weeks.^11^,^12^ Various inflammatory cells are involved in the different healing phases and are orchestrated by a large array of changing chemical signals. In a small previous study by Lapa et al., it could be shown that post-ischemic inflammation can be visualized by ^68^Ga-DOTATOC PET/CT within 3 to 10 days after myocardial infarction.^7^ However, further studies are needed to clarify, among others, which cell types are visualized in the different inflammatory phases and which subtypes of somatostatin receptors they express as well as what these findings have for a prognostic implication. This is inevitable to fully understand the diagnostic potential of this still young method for myocardial inflammation imaging. Despite the fact that larger knowledge gaps still exist, our case illustrates the importance of keeping an eye out for incidental cardiac findings on somatostatin receptor PET/CT in patients with clinically suspected NETs, as they might have important implications for the clinical decision-making process. Hereby, it should be kept in mind that false-positive myocardial uptake might occur due to the spillover from adjacent organs with high uptake such as the liver and spleen, which is particularly a potential problem in the inferior wall segments of the heart. Alternatively, cardiac uptake might also be due to a primary or secondary (metastatic) NET, which exhibits usually a distinct, well-defined uptake on somatostatin receptor PET/CT with varying degree of uptake intensity that depends on the degree of underlying tumor cell differentiation.^13^,^14^ In contrast, myocardial inflammation usually displays less well-defined areas with uptake of low intensity that follow either a coronary vessel territory, in case of post-ischemic inflammation, or exhibit typically multiple uptake areas, as in myocarditis or cardiac sarcoidosis.^7^ The subsequent differentiation of the underlying etiology solely based on somatostatin receptor PET/CT can be challenging. Therefore, additional investigations can be indicated, as for instance CMR with its multiparametric capabilities for tissue characterization, to ensure the correct diagnosis as well as initiation of appropriate treatment if necessary.

In our case, a series of unexpected findings were of great importance as they revealed the underlying cause of the patient’s symptoms as well as its subsequent complications. It is most likely that our patient had an undetected pheochromocytoma for many years, which is a catecholamine-secreting tumor arising from chromaffin cells of the adrenal medulla, which occurs in approximately 0.2%-0.5% of patients with hypertension.^4^,^15^ The patient’s longstanding, difficult to treat secondary hypertension resulted in concentric left ventricular hypertrophy and might have contributed in combination with other factors to the development of coronary artery disease with subsequent “silent” myocardial infarction. However, the patient’s myocardial infarction could have also been catecholamine-induced.^16^,^17^ The classic triad of symptoms in patients with pheochromocytoma consists of episodic headache, sweating, and tachycardia. Nonetheless, less common symptoms have been reported among others fever, which can be due to tumor necrosis as found in our patient.^18^ Furthermore, chronic catecholamine excess can lead to an increase in inflammatory markers, as in our case of the C-reactive protein.^19^ Rarely, pheochromocytoma can even cause cardiac arrhythmias or catecholamine-induced myocarditis as well as heart failure with subsequent pulmonary edema due to the catecholamine excess that is similar to a stress-induced (takotsubo) cardiomyopathy.^17^ This variety of potential cardiovascular effects of a pheochromocytoma should be kept in mind when interpreting a somatostatin receptor PET/CT, as several of them can lead to detectable myocardial inflammation. Another form of NET, commonly originating from the gastrointestinal tract and originally termed “carcinoid,” can lead to pathognomonic plaque like depositions in the endocardium of the valvular apparatus and ventricles (mainly in the right heart) as well as intima of the pulmonary arteries or aorta.^20^ The pathophysiology of this so-called carcinoid heart disease is poorly understood, and it is unclear whether inflammation can be visualized during the characteristic plaque formation process or not.

## Conclusion

Our case highlights the importance of paying attention to incidental cardiac findings on somatostatin receptor PET/CT in patients with clinically suspected NETs, as they cannot only indicate a primary or secondary (metastatic) NET, but also areas of myocardial inflammation, which is a potential driving force for cardiac remodeling and poor prognosis. Therefore, it is usually advisable to determine the underlying etiology of an eventual cardiac uptake with the help of additional investigations, as, for instance, CMR, to ensure the correct diagnosis as well as initiation of appropriate treatment if indicated.
